# Supplementary material for: MCM complexes are barriers that restrict cohesin-mediated loop extrusion
Source: Nature. 2022 May 18;606(7912):197–203. doi: 10.1038/s41586-022-04730-0 (PMC9159944; doi:10.1038/s41586-022-04730-0)
Supplement: Supplementary file 1 — This file contains Supplementary Figures 1-6. [file 41586_2022_4730_MOESM1_ESM.pdf]

nature portfolio

https://doi.org/10.1038/s41586-022-04730-0

Supplementary information

MCM complexes are barrier cohesin-mediated loop extr

In the format provided by the authors and unedited

Supplementary Figure 1 | Gel source data

a) Uncropped blots related to Extended Data Fig. 7c

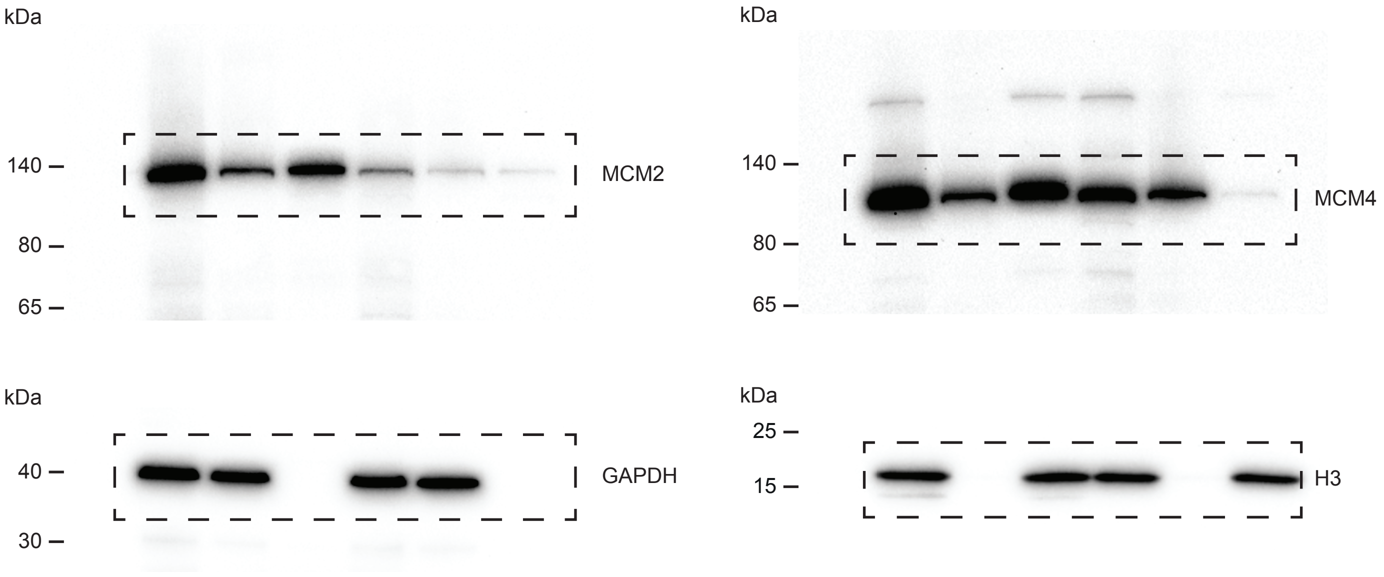

b) Uncropped blots related to Extended Data Fig. 8c

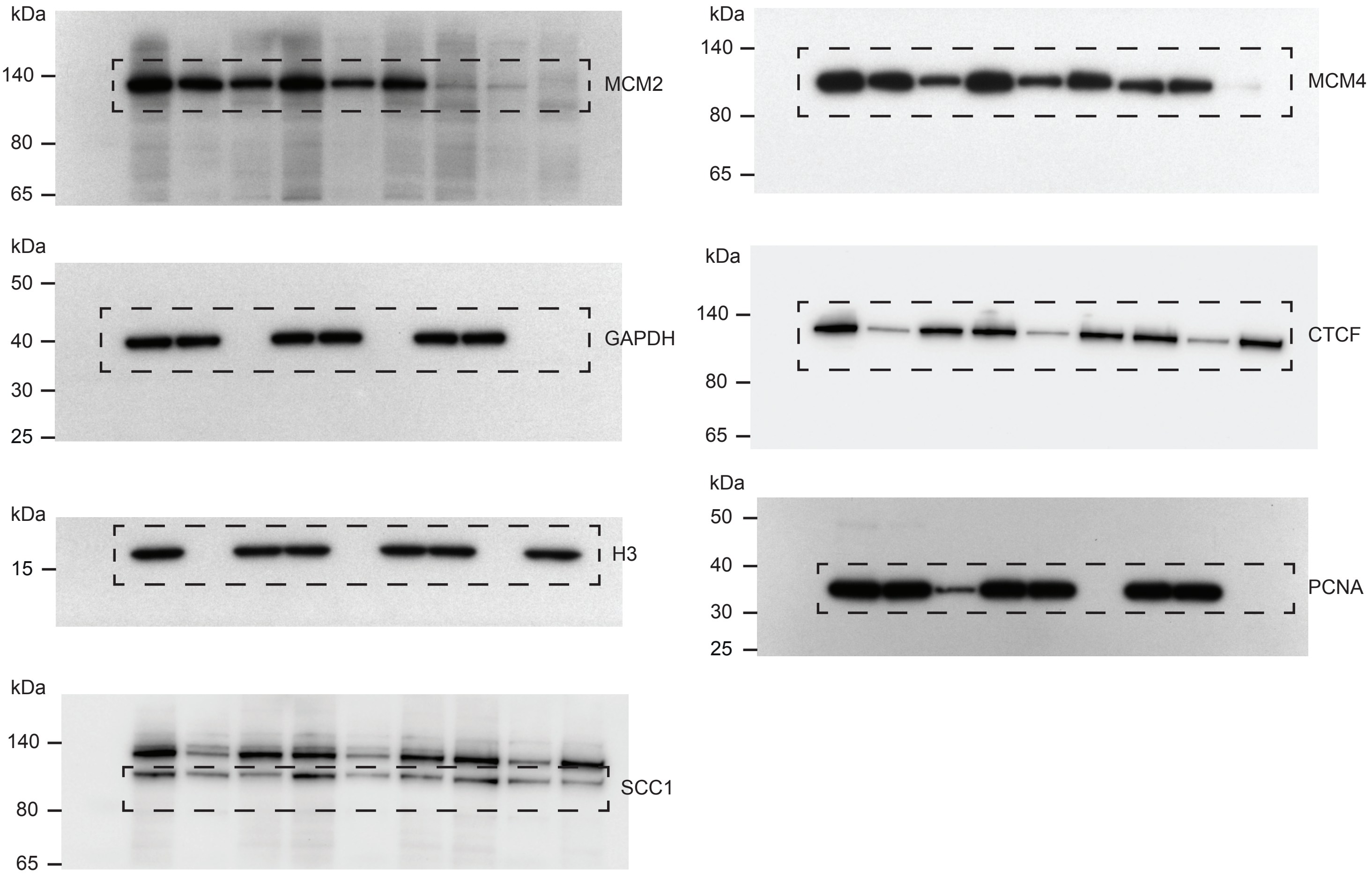

c) Uncropped blots related to Extended Data Fig. 8n

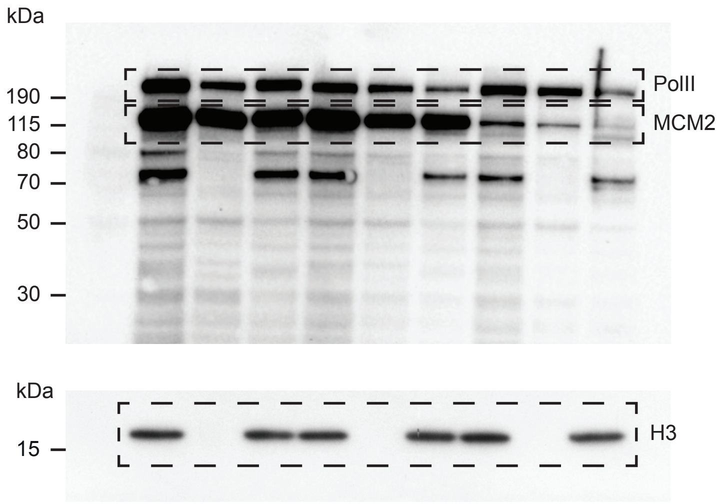

Supplementary Figure 2 | Parameter sweep for peak strengths for simulated paternal chromatin in wild type and MCM loss conditions

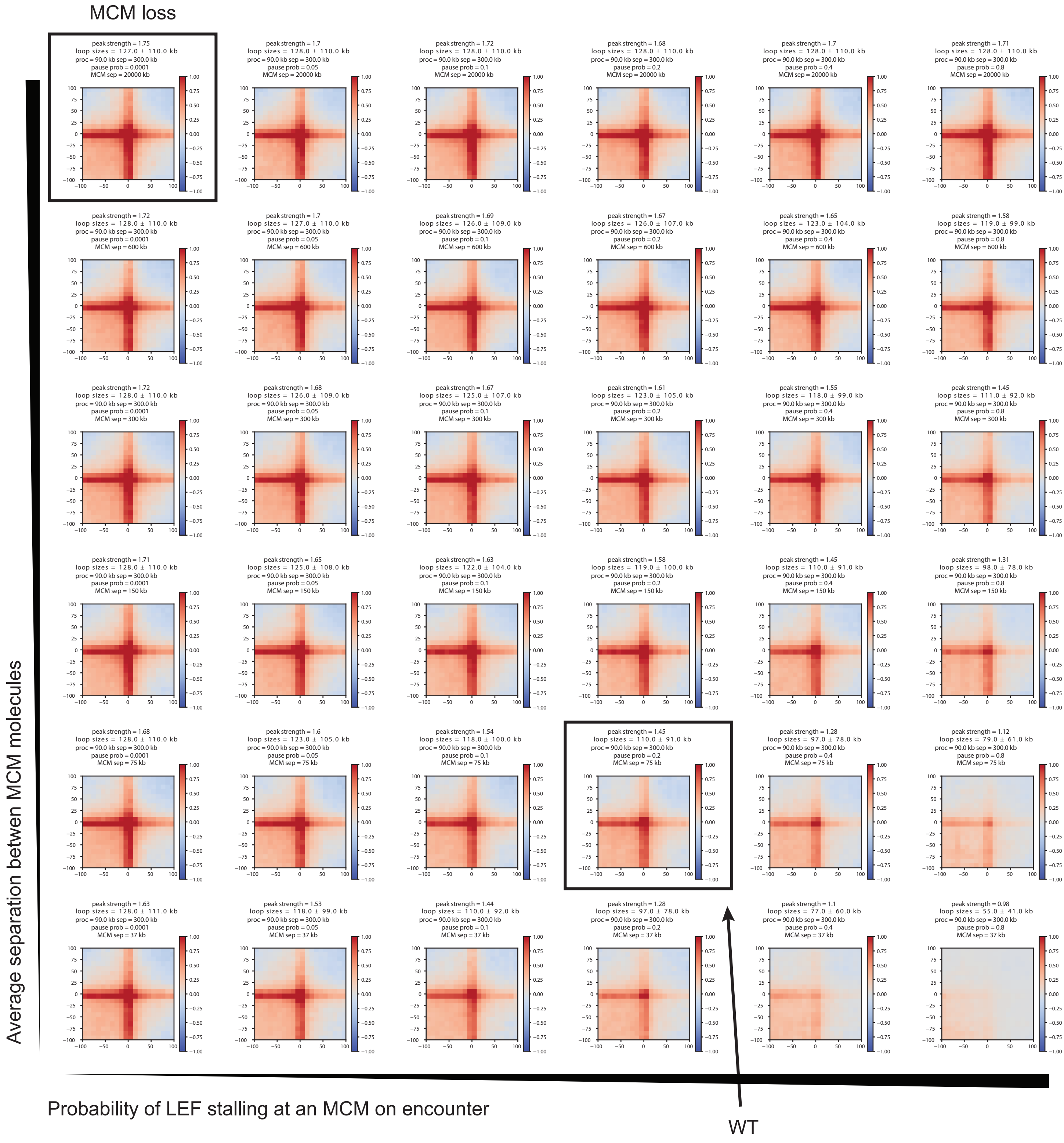

Supplementary Figure 3 | Parameter sweep for peak strengths for simulated paternal chromatin in *Wapl*$^{\Delta}$ and *Wapl*$^{\Delta}$/MCM loss conditions

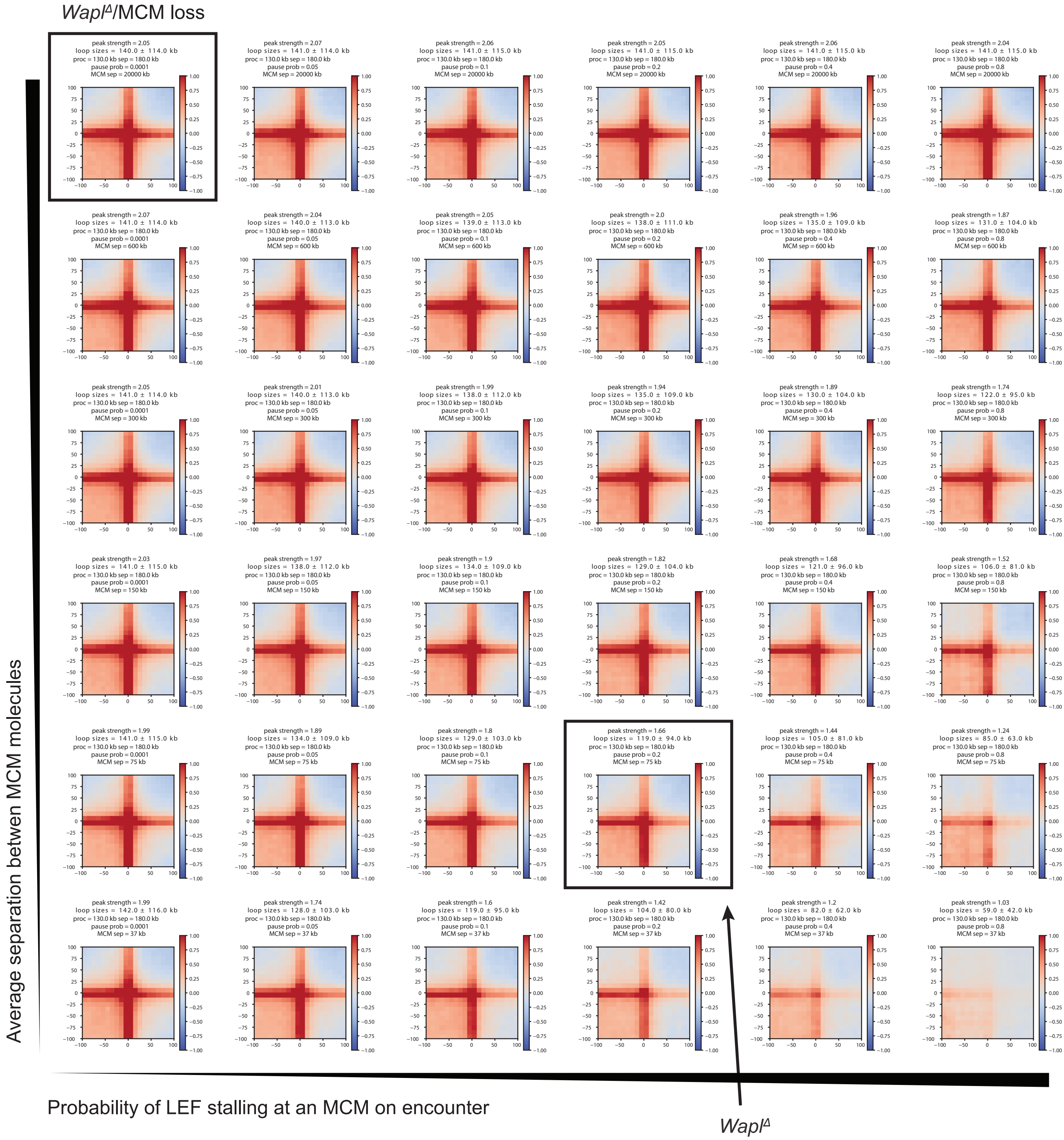

Supplementary Figure 4 | Parameter sweep for contact probability decay curves Pc(s) for simulated paternal chromatin in wild type and MCM loss conditions

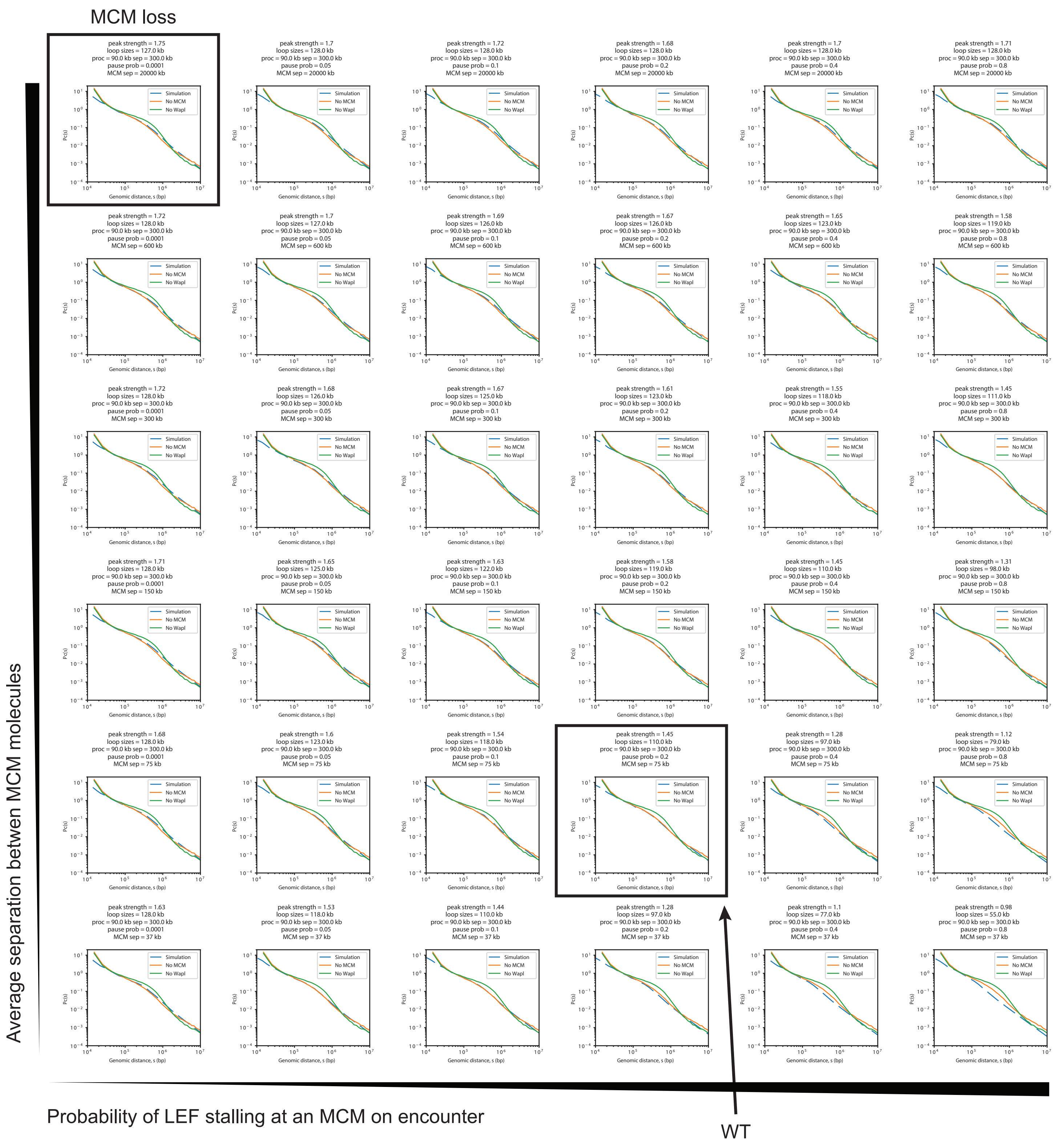

Supplementary Figure 5 | Parameter sweep for contact probability decay curves Pc(s) for simulated paternal chromatin in *Wapl*$^{\Delta}$ and *Wapl*$^{\Delta}$/MCM loss conditions

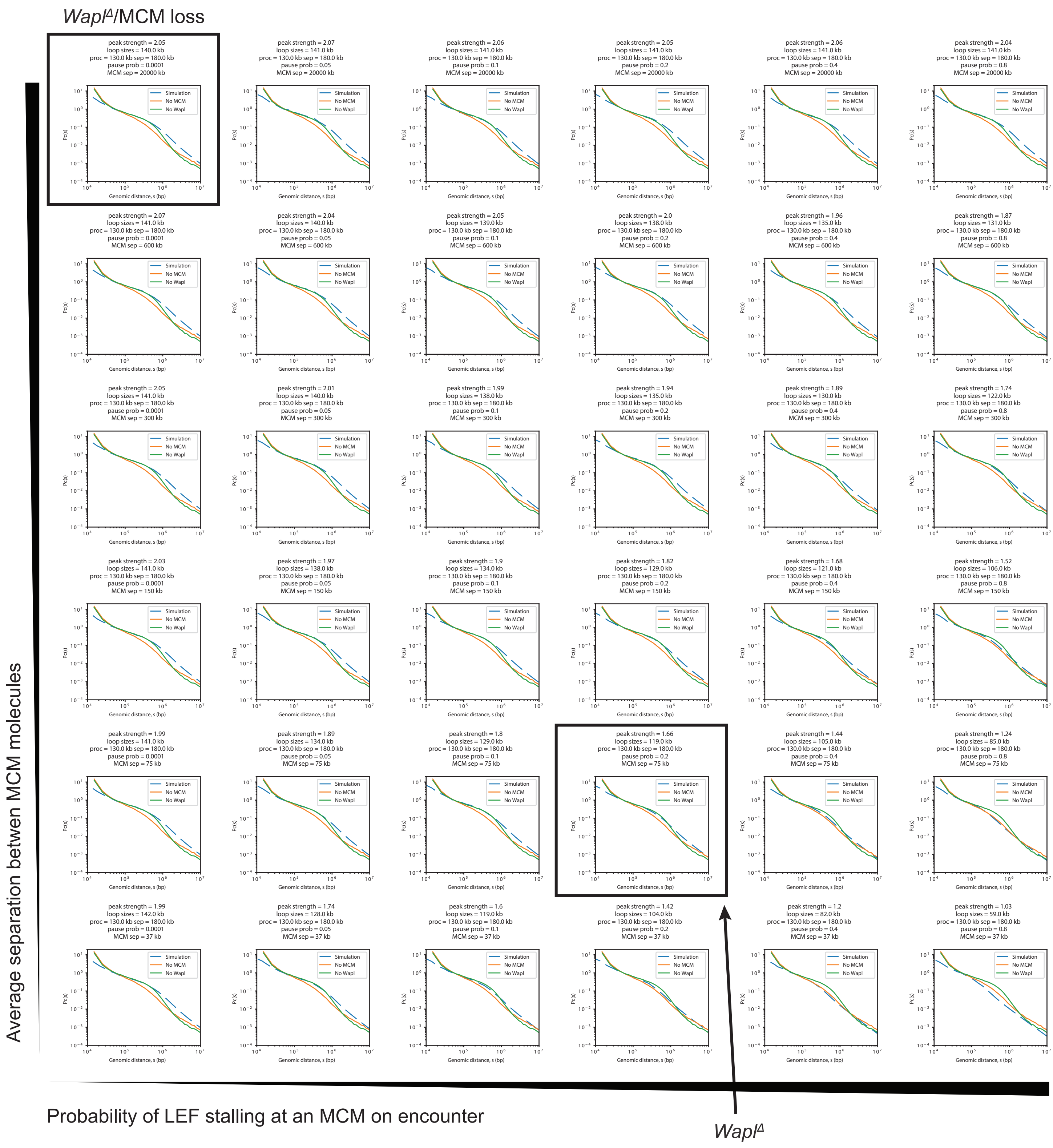

Supplementary Figure 6 | FACS gating strategy

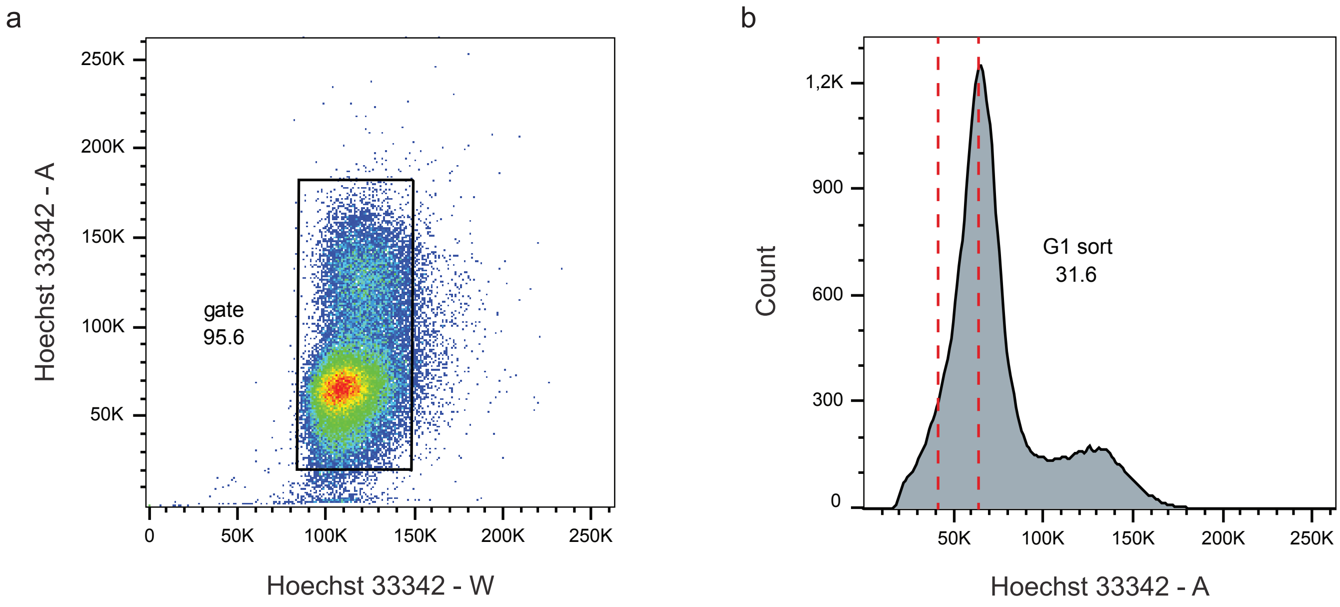

SI Guide

MCM complexes are barriers that restrict cohesin-mediated loop extrusion

1. SUPPLEMENTARY FIGURES

Supplementary Figure 1 | Gel source data. (a) Uncropped blots related to Extended Data Fig. 7c. The dashed black boxes indicate the displayed regions. GAPDH and H3 loading controls were run on the same gel, but blot was cut after transfer at 50 kDa and at 25 kDa. Staining for MCM2 and MCM4 was done on the same blot. (b) Uncropped blots related to Extended Data Fig. 8c. The dashed black boxes indicate the displayed regions. GAPDH and H3 loading controls were run on the same gel, but blot was cut after transfer at 50 kDa and at 25 kDa. Staining for MCM2, MCM4, CTCF and SCC1 was done on the same blot; PCNA-staining was done on the same blot as GAPDH. (c) Uncropped blots related to Extended Data Fig. 8n. H3 loading control was run on the same gel, but blot was cut after transfer at 25 kDa.

Supplementary Figure 2 | Parameter sweep for peak strengths for simulated paternal chromatin in wild type and MCM loss conditions.

Supplementary Figure 3 | Parameter sweep for peak strengths for simulated paternal chromatin in *Wapl*$^{\Delta}$ and *Wapl*$^{\Delta}$/MCM loss conditions.

Supplementary Figure 4 | Parameter sweep for contact probability decay curves $P_c(s)$ for simulated paternal chromatin in wild type and MCM loss conditions.

Supplementary Figure 5 | Parameter sweep for contact probability decay curves $P_c(s)$ for simulated paternal chromatin in *Wapl*$^{\Delta}$ and *Wapl*$^{\Delta}$/MCM loss conditions.

Supplementary Figure 6 | FACS gating strategy. (a) Hoechst-W vs Hoechst-A gate for one of the HCT116 MCM2-mAID samples synchronized in G1 to select the Hoechst 33342-stained cell population. The percentage of cells inside the gate is denoted in the graph. (b) Count vs Hoechst-A is shown for the cell population gated in panel (a) and generates a histogram of cell number in function of Hoechst 33342 intensity. To avoid S-phase cell contamination, only cells in the left part of the G1 peak were collected (red dashed box). The percentage of cells collected is denoted in the graph.

2. SUPPLEMENTARY TABLES

Supplementary Table 1. Read statistics of Hi-C and micro-C replicates.

Supplementary Table 2. Oligonucleotide sequences used for the DNA substrate for single-molecule imaging.

3. SUPPLEMENTARY VIDEOS

Supplementary Video 1. Video showing translocating cohesin (green) on doubly-tethered DNA (blue) in the absence of MCM at 0.15 M NaCl (corresponds to kymograph in Extended Data Fig. 9b).

Supplementary Video 2. Video showing origin-bound MCM (magenta) as an efficient barrier for cohesin translocation (green) on doubly-tethered DNA (blue) at 0.15 M NaCl (corresponds to kymograph in Fig. 4b).

Supplementary Video 3. Video showing translocating cohesin (green) on doubly-tethered DNA (blue) that occasionally can bypass origin-bound MCM (magenta) at 0.15 M NaCl (corresponds to kymograph in Extended Data Fig. 9e).

Supplementary Video 4. Video showing translocating cohesin (green) on doubly-tethered DNA (blue) in the absence of MCM at 0.5 M NaCl (corresponds to kymograph in Extended Data Fig. 10b).

Supplementary Video 5. Video showing origin-bound MCM (magenta) as an efficient barrier for cohesin translocation (green) on doubly-tethered DNA (blue) at 0.5 M NaCl (corresponds to kymograph in Extended Data Fig. 10c, left).

Supplementary Video 6. Video showing translocating cohesin (green) on doubly-tethered DNA (blue) that occasionally can bypass origin-bound MCM (magenta) at 0.5 M NaCl (corresponds to kymograph in Extended Data Fig. 10d, left).
