# Supplementary material for: MCM complexes are barriers that restrict cohesin-mediated loop extrusion
Source: Nature. 2022 May 18;606(7912):197–203. doi: 10.1038/s41586-022-04730-0 (PMC9159944; doi:10.1038/s41586-022-04730-0)
Supplement: Supplementary file 2 — Reporting Summary [file 41586_2022_4730_MOESM2_ESM.pdf]

nature research

Corresponding author(s): Kikuë Tachibana

Last updated by author(s): Mar 6, 2022

# Reporting Summary

Nature Research wishes to improve the reproducibility of the work that we publish. This form provides structure for consistency and transparency in reporting. For further information on Nature Research policies, see our Editorial Policies and the Editorial Policy Checklist.

## Statistics

For all statistical analyses, confirm that the following items are present in the figure legend, table legend, main text, or Methods section.

| n/a | Confirmed | |
|---|---|---|
| ☐ | ☒ | The exact sample size (*n*) for each experimental group/condition, given as a discrete number and unit of measurement |
| ☐ | ☒ | A statement on whether measurements were taken from distinct samples or whether the same sample was measured repeatedly |
| ☐ | ☒ | The statistical test(s) used AND whether they are one- or two-sided<br>*Only common tests should be described solely by name; describe more complex techniques in the Methods section.* |
| ☒ | ☐ | A description of all covariates tested |
| ☐ | ☒ | A description of any assumptions or corrections, such as tests of normality and adjustment for multiple comparisons |
| ☐ | ☒ | A full description of the statistical parameters including central tendency (e.g. means) or other basic estimates (e.g. regression coefficient) AND variation (e.g. standard deviation) or associated estimates of uncertainty (e.g. confidence intervals) |
| ☐ | ☒ | For null hypothesis testing, the test statistic (e.g. *F*, *t*, *r*) with confidence intervals, effect sizes, degrees of freedom and *P* value noted<br>*Give P values as exact values whenever suitable.* |
| ☒ | ☐ | For Bayesian analysis, information on the choice of priors and Markov chain Monte Carlo settings |
| ☒ | ☐ | For hierarchical and complex designs, identification of the appropriate level for tests and full reporting of outcomes |
| ☒ | ☐ | Estimates of effect sizes (e.g. Cohen's *d*, Pearson's *r*), indicating how they were calculated |

*Our web collection on statistics for biologists contains articles on many of the points above.*

## Software and code

Policy information about availability of computer code

| | |
|---|---|
| Data collection | - Images: LSM780 and LSM880 microscope using a plan-apochromat 63x/1.4 oil immersion objective operated by ZEN acquisition software ZEN Black (2.8) and ZEN Blue (3.0-3.3) (ZEISS)<br>- Sequencing data: HiSeq 2500 v4 system in PE125 mode, NextSeq system using high-output lane in PE75 mode, NovaSeq 6000 system in PE100 mode<br>- Western blotting: ChemiDoc imaging system operated by Image Lab software (Bio-Rad)<br>- Single-molecule assay: RM21 micromirror TIRF microscope (Mad City Labs) with an Apo N TIRF 60 x oil-immersion TIRF objective (NA 1.49, Olympus) |
| Data analysis | numpy (Version 1.19.5)<br>scipy (Version 1.4.1)<br>pandas (Version 1.3.4)<br>matplotlib (Version 3.1.3)<br>seaborn (Version 0.11.2)<br>pingouin (Version 0.3.12)<br>scikit_posthocs (Version 0.6.7)<br>pairtools (https://pairtools.readthedocs.io/en/latest/)<br>distiller (https://github.com/open2c/distiller-nf)<br>cooltools (https://github.com/open2c/cooltools)<br>chromosight (https://github.com/koszullab/chromosight)<br>coolpup.py (https://github.com/open2c/coolpuppy)<br>Mustache (https://github.com/ay-lab/mustache)<br>HICcompare (https://github.com/dozmorovlab/HiCcompare)<br>ImageJ/FIJI (Version 1.53c)<br>SVI Huygens Professional (Version 20.04) |

R (Version 3.6.3)
RStudio (Version 1.3.959)
ggplot2 (Version 3.3.3)
Kallisto (Version 0.46.1)
ShinyGO (http://bioinformatics.sdstate.edu/go/)
Molecule Archive Suite (Mars) plugin (https://github.com/duderstadt-lab/)
ImageLab (Version 5.2.1)
Prism (Version 8.3.1)
Microsoft Excel for Mac (Version 16.26)

For manuscripts utilizing custom algorithms or software that are central to the research but not yet described in published literature, software must be made available to editors and reviewers. We strongly encourage code deposition in a community repository (e.g. GitHub). See the Nature Research guidelines for submitting code & software for further information.

## Data

Policy information about availability of data

All manuscripts must include a data availability statement. This statement should provide the following information, where applicable:

- Accession codes, unique identifiers, or web links for publicly available datasets
- A list of figures that have associated raw data
- A description of any restrictions on data availability

All sequencing data in support of the findings of this study have been deposited in the Gene Expression Omnibus (GEO) under the series accession numbers GSE196497 (snHi-C and RNA-Seq) and GSE155971 (Hi-C and Micro-C). The single-molecule video datasets supporting the findings in this study are deposited at Zenodo with following DOI´s: 10.5281/zenodo.5911107 (high salt experiments), 10.5281/zenodo.5911211 (physiological salt experiments) and 10.5281/zenodo.5911285 (ydf experiments). All data are also available from the authors upon request.

# Field-specific reporting

Please select the one below that is the best fit for your research. If you are not sure, read the appropriate sections before making your selection.

☒ Life sciences ☐ Behavioural & social sciences ☐ Ecological, evolutionary & environmental sciences

For a reference copy of the document with all sections, see nature.com/documents/nr-reporting-summary-flat.pdf

# Life sciences study design

All studies must disclose on these points even when the disclosure is negative.

| | |
|---|---|
| Sample size | No statistical methods were used to estimate sample size. Sample size was determined based on previous studies in the field to generate reproducible results. |
| Data exclusions | Exclusion criteria for snHi-C data:<br>- Unfertilized cells, polyspermic cells and zygotes in wrong cell cycle phase were excluded from the snHi-C procedure.<br>- snHi-C samples with < 100k total contacts were excluded from all analyses. |
| Replication | snHi-C: Number of mice and pronuclei is indicated in the figure legends. In general, 3-4 independent experiments were performed using 3-6 females for each genotype.<br>RNA-Seq: RNA-Seq for G1 and G2 zygotes was performed in duplicate and RNA-Seq for tissue culture cells was performed in triplicate.<br>Hi-C/micro-C: Hi-C/micro-C experiments with the MCM2-mAID cell line were performed using 1 Hi-C replicate and 2 biological independent micro-C replicates for the condition DMSO vs AUX and 1 micro-C replicate for the condition DMSO/trp vs AUX/trp.<br>Single-molecule studies: Number of observations analyzed is indicated in the figure or figure legends. All single-molecule observations were derived from at least three independent experiments.<br><br>All attempts for replication were successful. |
| Randomization | Randomization was not relevant to this study. Samples were organized into identifiable groups based on the experimental conditions (e.g. wild type vs mutant mouse strain, +/- gemininL26A expression in G1 zygotes, auxin vs dmso treatment in HCT116 MCM2-mAID cell line, cohesin pausing at MCM or MCM-Mcm3-YDF). Samples within control or experimental groups were randomly assigned. |
| Blinding | Blinding was not relevant due to the design of this work. The researchers need to verify the experimental conditions (e.g. wild type vs mutant mouse strain, experimental time points, auxin vs dmso treatment in HCT116 MCM2-mAID cell line). Observer bias was prevented because the samples were measured using DNA sequencing or automated microscopy and the analysis was performed using automated python and R scripts. |

# Reporting for specific materials, systems and methods

We require information from authors about some types of materials, experimental systems and methods used in many studies. Here, indicate whether each material, system or method listed is relevant to your study. If you are not sure if a list item applies to your research, read the appropriate section before selecting a response.

## Materials & experimental systems

| n/a | Involved in the study | |
| --- | --- | --- |
| ☐ | ☒ | Antibodies |
| ☐ | ☒ | Eukaryotic cell lines |
| ☒ | ☐ | Palaeontology and archaeology |
| ☐ | ☒ | Animals and other organisms |
| ☒ | ☐ | Human research participants |
| ☒ | ☐ | Clinical data |
| ☒ | ☐ | Dual use research of concern |

## Methods

| n/a | Involved in the study | |
| --- | --- | --- |
| ☒ | ☐ | ChIP-seq |
| ☐ | ☒ | Flow cytometry |
| ☒ | ☐ | MRI-based neuroimaging |

# Antibodies

Antibodies used

For immunofluorescence in oocytes and zygotes:
Primary antibodies
- anti-MCM2 (1:500, BD transduction Laboratories, #610701)
- anti-CTCF (1:250, Peters laboratory A992)
- anti-MYC (1:500, Millipore, #05-724)
Secondary antibodies
- Goat anti-mouse Alexa Fluor 488 (1:500, Invitrogen, #A11029)
- Donkey anti-rabbit Alexa Fluor 568 (1:500, Invitrogen, #A10042)
- Goat anti-mouse Alexa Fluor 647 (1:500, Invitrogen, #A-21235)

For Western blotting:
Primary antibodies
- anti-MCM2 (1:5000, BD transduction Laboratories, #610701)
- anti-MCM4 (1:5000, Abcam, #ab4459)
- anti-H3 (1:2000, Cell Signaling, #9715S)
- anti-GAPDH (1:2500, Millipore, #MAB374)
- anti-CTCF (1:1000, Peters laboratory A992)
- anti-PCNA (1:500, Santa Cruz, #PC10)
- anti-SCC1 (1:1000, Millipore, #05-908)
- anti-Pol II 8WG16 (1:500, Santa Cruz, #sc-56767)
Secondary antibodies:
- Goat Anti-Mouse Immunoglobulins/HRP (1:500, Dako, #P0447)
- Goat Anti-Rabbit Immunoglobulins/HRP (1:500, Dako, #P0448)

Validation

- anti-MCM2: https://www.bdbiosciences.com/us/reagents/research/antibodies-buffers/cell-biology-reagents/cell-biology-antibodies/purified-mouse-anti-bm28-46bm28/p/610701
- anti-CTCF: Wutz, G. et al. Topologically associating domains and chromatin loops depend on cohesin and are regulated by CTCF, WAPL, and PDS5 proteins. The EMBO journal e201798004 (2017)
- anti-MCM4: https://www.abcam.com/mcm4-antibody-ab4459.html
- anti-MYC: https://www.merckmillipore.com/BE/fr/product/Anti-Myc-Tag-Antibody-clone-4A6,MM_NF-05-724?ReferrerURL=https%3A%2F%2Fwww.google.com%2F
- anti-H3: https://www.cellsignal.com/products/primary-antibodies/histone-h3-antibody/9715?Ntk=Products&Ntt=9715
- anti-GAPDH: https://www.merckmillipore.com/AT/de/product/Anti-Glyceraldehyde-3-Phosphate-Dehydrogenase-Antibody-clone-6C5,MM_NF-MAB374?ReferrerURL=https%3A%2F%2Fwww.google.com%2F
- anti-SCC1: https://www.merckmillipore.com/BE/fr/product/Anti-RAD21-Antibody,MM_NF-05-908?ReferrerURL=https%3A%2F%2Fwww.google.com%2F&bd=1
- anti PCNA: https://www.scbt.com/p/pcna-antibody-pc10
- anti-Pol II 8WG16: https://www.scbt.com/p/pol-ii-antibody-f-12
gclid=EAIaIQobChMIw-3uuK6g9AIVjLh3Ch0lKg_6EAAYASAAEgKMiPD_BwE
- Alexa Goat anti-mouse 488: https://www.thermofisher.com/antibody/product/Goat-anti-Mouse-IgG-H-L-Highly-Cross-Adsorbed-Secondary-Antibody-Polyclonal/A-11029
- Alexa Donkey anti-rabbit 568: https://www.thermofisher.com/antibody/product/Donkey-anti-Rabbit-IgG-H-L-Highly-Cross-Adsorbed-Secondary-Antibody-Polyclonal/A10042
- Alexa Goat anti-mouse 647: https://www.thermofisher.com/antibody/product/Goat-anti-Mouse-IgG-H-L-Cross-Adsorbed-Secondary-Antibody-Polyclonal/A-21235
- Goat Anti-Mouse Immunoglobulins/HRP: https://www.agilent.com/en/product/immunohistochemistry/antibodies-controls/secondary-antibodies/goat-anti-mouse-immunoglobulins-hrp-(affinity-isolated)-153239
- Goat Anti-Rabbit Immunoglobulins/HRP: https://www.agilent.com/en/product/immunohistochemistry/antibodies-controls/secondary-antibodies/goat-anti-rabbit-immunoglobulins-hrp-(affinity-isolated)-153244

# Eukaryotic cell lines

Policy information about cell lines

Cell line source(s)

HCT116 MCM2-mAID-NeoR (biallelic), CMV-AtAFB2
Clone 16

| | |
|---|---|
| | Supplied by Toyoaki Natsume<br>Described in: Natsume, T. et al. Acute inactivation of the replicative helicase in human cells triggers MCM8-9-dependent DNA synthesis. Genes & development 31, 816–829 (2017). |
| Authentication | HR-mediated C-terminal tagging of MCM2 in the HCT116 MCM2-mAID-NeoR cell line was examined by western blotting. No growth defects were observed in this clone. |
| Mycoplasma contamination | Cell line tested negative for mycoplasma test. |
| Commonly misidentified lines<br>(See ICLAC register) | No commonly misidentified cell line was used. |

## Animals and other organisms

Policy information about studies involving animals; ARRIVE guidelines recommended for reporting animal research

| | |
|---|---|
| Laboratory animals | Mice were housed in individually ventilated cages under a 14h light/10h dark cycle at ambient temperature of 22 °C ± 1 °C and humidity of 55% ± 5% with continuous access to food and water. Animals were housed grouped (maximum 4 males per cage and maximum 5 females per cage). All mice were bred in the IMBA animal facility. Wildtype, Scc1fl/fl and Scc1Myc/+ mice were bred on a mixed background (B6, 129, Sv). Waplfl/fl and Zp3-dsCTCF mice were bred on a primarily C57BL/6J background. Zp3-dsCTCF mice were maintained by breeding Zp3-dsCTCF males to C57BL/6J females. Experimental Scc1fl/fl and Waplfl/fl mice were obtained by mating of homozygous floxed females to homozygous floxed males carrying Tg(Zp3Cre). Experimental Scc1Myc/+ mice were obtained by intercrossing heterozygous Scc1Myc/+ mice. Experimental Zp3-dsCTCF mice were maintained by breeding Zp3-dsCTCF males to C57BL/6J females. Experimental females/males were between 2 to 5 months old. |
| Wild animals | This study did not involve wild animals. |
| Field-collected samples | This study did not involve samples collected from the field. |
| Ethics oversight | The mice used in this work were bred and maintained in agreement with the authorizing committee according to the Austrian Animal Welfare law and the guidelines of the International Guiding Principles for Biomedical Research Involving Animals (CIOMS, the Council for International Organizations of Medical Sciences). |

Note that full information on the approval of the study protocol must also be provided in the manuscript.

## Flow Cytometry

### Plots

Confirm that:

☒ The axis labels state the marker and fluorochrome used (e.g. CD4-FITC).

☒ The axis scales are clearly visible. Include numbers along axes only for bottom left plot of group (a 'group' is an analysis of identical markers).

☒ All plots are contour plots with outliers or pseudocolor plots.

☒ A numerical value for number of cells or percentage (with statistics) is provided.

### Methodology

| | |
|---|---|
| Sample preparation | For G1 FACS-sorting, cells were synchronized with a double-thymidine arrest–release followed by release into fresh medium for 12 h. 4 h before sorting Hoechst 33342 (Sigma) was added to the medium at a concentration of 0.2 µg/ml. After harvesting, the cells were resuspended in FACS-buffer (PBS + 2% FCS) and immediately sorted. |
| Instrument | BD FACSAria™ III Cell Sorter |
| Software | The BD FACSAria™ III Cell Sorter controlled by FACSDiva software. Flow cytometry data was analyzed using FlowJo V10. |
| Cell population abundance | 1 million HCT116 MCM2-mAID cells were sorted for each sample. |
| Gating strategy | SSC-A vs FSC-A gating was used to filter out debris followed by FSC-H vs FSC-A gating to exclude doublets. The cells stained with Hoechst 33342 were selected using HOECHST-W vs HOECHST-A gating and the G1 population could be selected with Count vs HOECHST-A gating. To avoid S-phase cell contamination, only cells in the left part of the G1 peak were collected (red dashed box in Extended Data Fig. 8b). |

☒ Tick this box to confirm that a figure exemplifying the gating strategy is provided in the Supplementary Information.
